# Association between atherogenic index of plasma and new-onset stroke in individuals with different glucose metabolism status: insights from CHARLS

**DOI:** 10.1186/s12933-024-02314-y

**Published:** 2024-06-21

**Authors:** Longjie Qu, Shuang Fang, Zhen Lan, Shuai Xu, Jialiu Jiang, Yilin Pan, Yun Xu, Xiaolei Zhu, Jiali Jin

**Affiliations:** 1 Department of Neurology, Nanjing Drum Tower Hospital, Clinical College of Nanjing Medical University, Nanjing, 210008 China; 2 Department of Neurology, Nanjing Drum Tower Hospital, Affiliated Hospital of Medical School, Nanjing University, Nanjing, 210008 China; 3 State Key Laboratory of Pharmaceutical Biotechnology, Institute of Translational Medicine for Brain Critical Diseases, Nanjing University, Nanjing, 210008 China; 4 Jiangsu Key Laboratory for Molecular Medicine, Medical School of Nanjing University, Nanjing, 210008 China; 5 Nanjing Neurology Clinical Medical Center, Nanjing, 210008 China

**Keywords:** Atherogenic index of plasma, Stroke, Pre-diabetes, Diabetes mellitus

## Abstract

**Background:**

Circulating atherogenic index of plasma (AIP) levels has been proposed as a novel biomarker for dyslipidemia and as a predictor of insulin resistance (IR) risk. However, the association between AIP and the incidence of new-onset stroke, particularly in individuals with varying glucose metabolism status, remains ambiguous.

**Methods:**

A total of 8727 participants aged 45 years or older without a history of stroke from the China Health and Retirement Longitudinal Study (CHARLS) were included in this study. The AIP was calculated using the formula log [Triglyceride (mg/dL) / High-density lipoprotein cholesterol (mg/dL)]. Participants were divided into four groups based on their baseline AIP levels: Q1 (AIP ≤ 0.122), Q2 (0.122 < AIP ≤ 0.329), Q3 (0.329 < AIP ≤ 0.562), and Q4 (AIP > 0.562). The primary endpoint was the occurrence of new-onset stroke events. The Kaplan–Meier curves, multivariate Cox proportional hazard models, and Restricted cubic spline analysis were applied to explore the association between baseline AIP levels and the risk of developing a stroke among individuals with varying glycemic metabolic states.

**Results:**

During an average follow-up of 8.72 years, 734 participants (8.4%) had a first stroke event. The risk for stroke increased with each increasing quartile of baseline AIP levels. Kaplan–Meier curve analysis revealed a significant difference in stroke occurrence among the AIP groups in all participants, as well as in those with prediabetes mellitus (Pre-DM) and diabetes mellitus (DM) (all P values < 0.05). After adjusting for potential confounders, the risk of stroke was significantly higher in the Q2, Q3, and Q4 groups than in the Q1 group in all participants. The respective hazard ratios (95% confidence interval) for stroke in the Q2, Q3, and Q4 groups were 1.34 (1.05–1.71), 1.52 (1.19–1.93), and 1.84 (1.45–2.34). Furthermore, high levels of AIP were found to be linked to an increased risk of stroke in both pre-diabetic and diabetic participants across all three Cox models. However, this association was not observed in participants with normal glucose regulation (NGR) (*p* > 0.05). Restricted cubic spline analysis also demonstrated that higher baseline AIP levels were associated with higher hazard ratios for stroke in all participants and those with glucose metabolism disorders.

**Conclusions:**

An increase in baseline AIP levels was significantly associated with the risk of stroke in middle-aged and elderly individuals, and exhibited distinct characteristics depending on the individual’s glucose metabolism status.

**Supplementary Information:**

The online version contains supplementary material available at 10.1186/s12933-024-02314-y.

## Introduction

Stroke is a major global public health burden with high morbidity and mortality. Despite diligent primary prevention efforts, the prevalence and incidence of stroke in China continue to exhibit an alarming increase. Consequently, it is imperative to develop low-cost and reproducible indicators that can enhance early identification of high-risk individuals with stroke [1]. DM and arterial hypertension are widely recognized as the most common risk factors for stroke [2]. Many studies have elucidated that metabolic disorders, including dyslipidemia and hyperglycemia, are significant risk factors for stroke. Several metabolic indicators, such as remnant cholesterol and triglyceride-glucose index, have been utilized to assess the risk and prognosis of stroke [3–5], but the predictive accuracy is still limited. The AIP, a logarithmically transformed ratio of fasting triglyceride to fasting high-density lipoprotein cholesterol, is a sensitive marker of lipoprotein profiles primarily reflecting plasma lipid levels. Recent researches suggest that rising AIP can indicate the severity of IR and is closely related to the development of IR and type 2 diabetes [6–8]. As a robust biomarker of dyslipidemia, AIP has been considered to be a powerful independent predictor of adverse cardiovascular and cerebrovascular events. AIP potentially serves as a significant predictor of intracranial arterial stenosis, the risk of ischemic stroke, and poor stroke outcomes.

[9–12]. On the other hand, AIP serves as a reliable indicator of IR, which indicates that it may differentiate stroke risk in individuals with abnormal glucose metabolism. However, the relationship between AIP and stroke based on an individual’s glucose metabolism status has been poorly investigated. Therefore, prospective cohort studies with a large sample size are warranted to clarify the relationship.

In the present study, we used data from the CHARLS to explore the association between baseline AIP levels and stroke under different glucose metabolic states.

## Methods

### Study participants

**Fig. 1:**
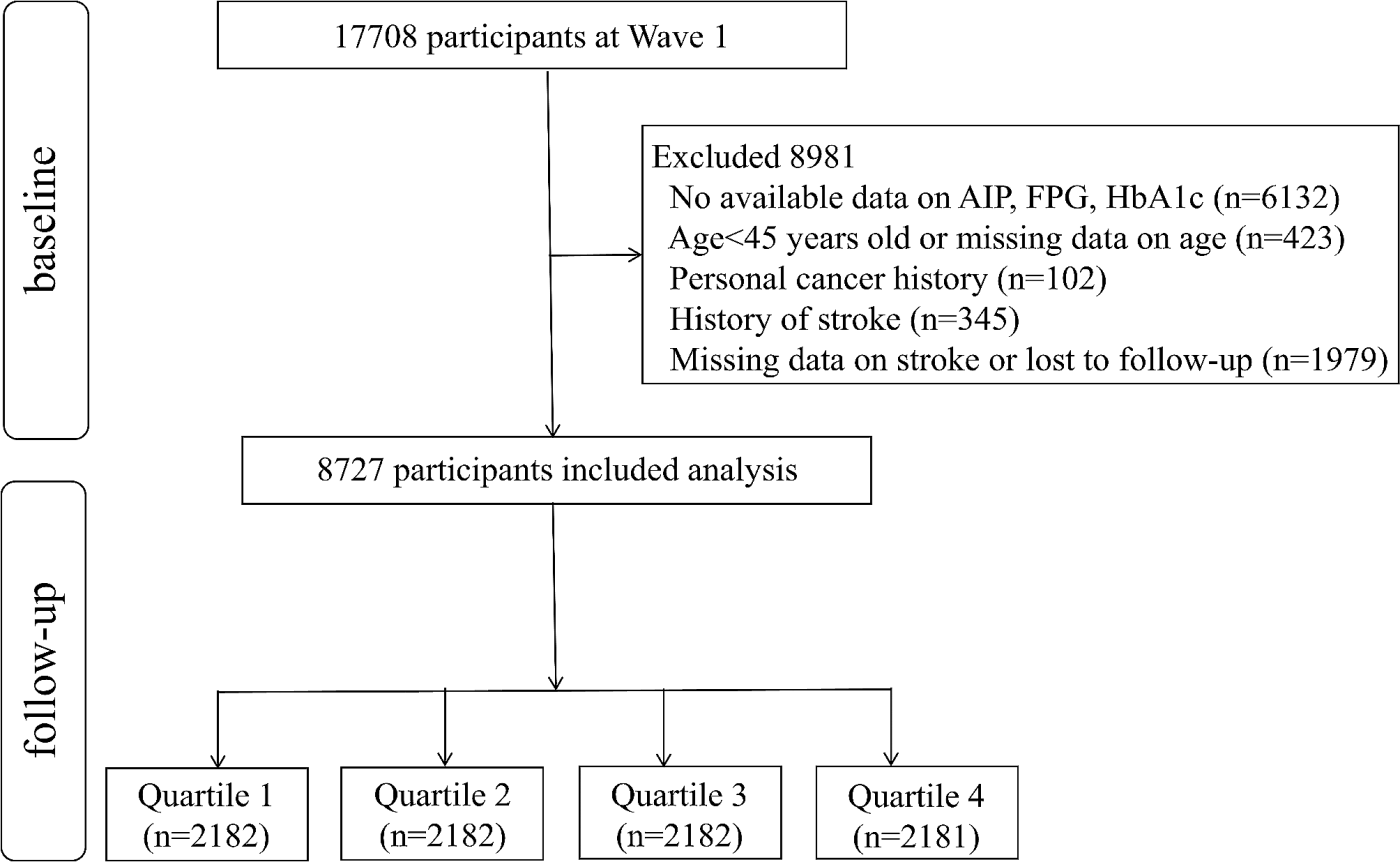
The flowchart of study participants

This study employed a prospective study design andall participants were derived from the CHARLS, a national cohort commenced in 2011. The cohort specifically focuses on individuals aged 45 years and older in China. The participants are traced once every 2–3 years to identify their health status. To date, five-wave periods of follow-up surveys have been completed, with data collected in 2011, 2013, 2015, 2018, and 2020. The detailed research methods have been described previously [13]. We initially enrolled 17,708 participants in CHARLS wave 1. Among these people, 8981 were excluded for meeting the following exclusion criteria: (1) missing available data on AIP, fasting blood glucose (FPG), glycosylated hemoglobin (HbA1c) (*n* = 6132), (2) age < 45 years old or missing data on age (*n* = 423), (3) personal cancer history (*n* = 102), (4) history of stroke (*n* = 345), (5) lack of data on stroke or lost to follow-up (*n* = 1979). Finally, 8727 participants were divided into four groups according to the baseline AIP quartiles and were followed up until 2020 (Fig. 1). The CHARLS study received ethical approval from the Biomedical Ethics Review Committee of Peking University (IRB00001052-11015), and all participants provided their written informed consent.

### Data collection

Trained interviewers collected the demographic information (such as age, sex, and marital status), health status and functioning (such as smoking, drinking, hypertension, and diabetes) of the participants with standard questionnaires. After a 15-minute rest, participants, except those with an arm injury, were instructed to undergo three left arm blood pressure measurements at a 45-second interval, and the average of three values was reported. Participants were asked to take off shoes, heavy clothes before weighed and measured for height. Body weight and height were measured using standardized scales to the nearest 0.1 kg and 0.1 cm, respectively. Venous blood samples were collected from each participant by medically trained staff following a standard phlebotomy protocol and assayed for biochemical measurements. Regrettably, nearly 8% of the participants who gave blood reported that they fasted less than 8 h before blood collection. The enzymatic colorimetric method was applied to measure FPG levels and serum lipid parameters, while HbA1c was measured using boronate affinity high performance liquid chromatography [14].

### Definitions

Hypertension diagnosis was based on a self-reported physician-diagnosed, and/or any antihypertensive medication use, and/or an average systolic/diastolic blood pressure (SBP/DBP) ≥ 140/90 mmHg [15]. DM was defined as FPG ≥ 126 mg/dl or HbA1c ≥ 6.5%, and/or a self-reported physician-diagnosed, and/or taking hypoglycemic agents. Pre-DM was characterized by an FPG of 100 to 125 mg/dL or an HbA1c of 5.7–6.4%. Individuals who were without DM or Pre-DM were classified as having NGR [16]. Dyslipidemia was diagnosed by a self-reported physician-diagnosed, and/or current use of lipid-lowering drugs, and/or total cholesterol (TC) ≥ 240 mg/dl, triglyceride (TG) ≥ 150 mg/dl, high-density lipoprotein cholesterol (HDL-C) < 40 mg/dl, low-density lipoprotein cholesterol (LDL-C) ≥ 160 mg/dl [17]. Cancer and heart disease were determined by participants’ self-reported. Body mass index (BMI) was calculated as the following formula: weight/height^2^ (kg/m^2^). The AIP was calculated as log (TG/HDL-C) [18].

### Follow up of endpoint events

The primary focus of the study was on the occurrence of the first stroke, which included both cerebral infarction and cerebral hemorrhage. Self-reported stroke was assessed with the following questions: “Have you been diagnosed with stroke by a doctor”; “Have you been diagnosed with stroke by a doctor since the last follow-up visit?”; “Compared to when we interviewed you last time, is your stroke condition better, about the same as it was then, or worse?”. The time of stroke events was determined by participants’ responses to questions: “When was the stroke first diagnosed or known by yourself?”; “When was your most recent stroke?”. All participants were followed up through five-wave interviews conducted from 2011 to either the occurrence of a stroke or 2020, whichever came first.

### Statistical analysis

Normally distributed continuous data were presented as mean ± standard deviation and analyzed for statistical significance using a one-way ANOVA. Non-normally distributed continuous data were expressed as median and interquartile range and analyzed by the Kruskal–Wallis test. Categorical data were described with counts and percentages and assessed using the chi-square test. Missing data of the participants included in this study were presented in Table S1 (see Additional file), and the multiple imputations method was applied to impute the missing data, assuming that the data was randomly missing.

Participants were divided into four groups according to the quartile level of the AIP: Quartile 1 (Q1), AIP ≤ 0.122; Quartile 2 (Q2), 0.122 < AIP ≤ 0.329; Quartile 3 (Q3), 0.329 < AIP ≤ 0.562; Quartile 4 (Q4), AIP > 0.562. In addition to its use as a categorical variable, AIP was also analyzed as a continuous variable to enhance the reliability of the results. Based on AIP grouping, the Kaplan–Meier method was used to estimate the cumulative incidence of stroke, and differences were compared using the log-rank test. Cox proportional hazard regression analyses were conducted to investigate the relationship between baseline AIP and the occurrence of stroke and to calculate the hazard ratio (HR) and 95% confidence interval (CI). Before analyzing, the assumption of proportional hazards was visually assessed by calculating the schoenfeld residuals. Three models were estimated: Model 1 applied an unadjusted model to estimate crude HR; Model 2 included adjustments for age, gender, marital status, drinking, smoking, residence, SBP, DBP, and BMI; Model 3 included adjustments for the variables in Model 2 as well as history of hypertension and heart disease, TC, FPG, HbA1c. The quartile 1 group was set as the reference in all models. In addition, restricted cubic splines analysis based on multivariable-adjusted Cox regression was conducted to visualize the linear or nonlinear relationship between baseline AIP levels and the risk of stroke. Moreover, to determine the prognostic value of AIP for stroke in different glucose metabolic states, we analyzed participants with NGR, Pre-DM, and DM, respectively. Subgroup analyses were stratified by baseline age (< 60 and ≥ 60 years), gender, BMI (< 24 and ≥ 24 kg/m^2^), residence (rural and urban), hypertension, and glucose metabolic states (NGR, Pre-DM, and DM) to assess the consistency of the adverse effect of AIP on new-onset stroke. To ensure the reliability of our findings, we performed two additional sensitivity analyses. Firstly, participants who had fasted for less than 8 h before blood collection were excluded. Secondly, we excluded participants with missing data for SBP, DBP, HR and BMI.

All statistical analyses were performed using IBM SPSS Statistics (Version 26), R (Version 4.3.2) and Rstudio (Version 1.3.1093). A two-sided P-value < 0.05 was considered to indicate statistical significance in the present study.

## Results

### General characteristics of participants

The baseline clinical and demographic characteristics of participants grouped by AIP quartile were presented in Table 1. The average age of participants at baseline was 58.04 ± 8.75 years, with 4742 (54.3%) being female. Participants in higher AIP quartiles tended to be younger, female, married, and better-educated compared to those in the lowest quartile. Additionally, they had lower proportions of rural residents, current smokers, and current alcohol consumers. The prevalence of hypertension, diabetes, dyslipidemia, and heart disease was higher among those in higher AIP quartiles. Moreover, SBP, DBP, heart rate, BMI, FPG, HbA1c, TC, TG, and LDL levels were elevated, while HDL-C levels were lower in these groups. The general characteristics were compared between the included and excluded participants in Table S2 (see Additional file). No significant differences were observed in hypertension, FPG, and TG between the included and excluded subjects.

**Table 1:** Baseline characteristics of participants categorized by AIP quartiles

Characteristics	Total	Quartiles of AIP				*P* value
		Q1	Q 2	Q 3	Q 4	
No. of participants	8727	2182	2182	2182	2181	
Age, years	58.04 ± 8.75	58.42 ± 8.95	58.18 ± 8.91	57.93 ± 8.62	57.63 ± 8.49	0.021
Female, n (%)	4742 (54.3)	1081 (49.5)	1208 (55.4)	1252 (57.4)	1201 (55.1)	< 0.001
SBP, mmHg	129.40 ± 20.01	126.21 ± 19.35	127.75 ± 19.52	131.07 ± 20.53	132.58 ± 20.01	< 0.001
DBP, mmHg	75.46 ± 11.37	73.30 ± 11.23	74.40 ± 10.93	76.43 ± 11.46	77.73 ± 11.33	< 0.001
Heart rate, bpm	71.95 ± 9.84	70.85 ± 9.73	71.50 ± 9.83	72.22 ± 9.72	73.22 ± 9.92	< 0.001
BMI, kg/m^2^	23.52 ± 3.35	21.98 ± 2.89	23.00 ± 3.17	24.11 ± 3.33	25.00 ± 3.22	< 0.001
Rural residence, n (%)	5755 (65.9)	1596 (73.1)	1486 (68.1)	1380 (63.2)	1293 (59.3)	< 0.001
Married, n (%)	7337 (84.1)	1815 (83.2)	1814 (83.1)	1829 (83.8)	1879 (86.2)	
Education, n (%)						0.007
Junior high school and below	7839 (89.8)	1996 (91.5)	1971 (90.3)	1956 (89.6)	1916 (87.8)	
Senior high school	788 (9.0)	169 (7.7)	186 (8.5)	201 (9.2)	232 (10.6)	
Tertiary	100 (1.1)	17 (0.8)	25 (1.1)	25 (1.1)	33 (1.5)	
Smoking, n (%)						
Never	5400 (61.9)	1280 (58.7)	1363 (62.5)	1392 (63.8)	1365 (62.6)	0.001
Former	793 (9.1)	194 (8.9)	181 (8.3)	204 (9.3)	214 (9.8)	
Current	2534 (29.0)	708 (32.4)	638 (29.2)	586 (26.9)	602 (27.6)	
Drinking, n (%)						< 0.001
Never	5143 (58.9)	1155 (52.9)	1310 (60.0)	1332 (61.0)	1346 (61.7)	
Former	668 (7.7)	137 (6.3)	171 (7.9)	207 (9.5)	153 (7.0)	
Current	2916 (33.4)	890 (40.8)	701 (32.1)	643 (29.5)	682 (31.3)	
Hypertension, n (%)	3377 (38.7)	664 (30.4)	735 (33.7)	931 (42.7)	1047 (48.0)	< 0.001
Diabetes, n (%)	1216 (13.9)	155 (7.1)	217 (9.9)	282 (12.9)	562 (25.8)	< 0.001
Dyslipidemia, n (%)	4230 (48.5)	314 (14.4)	536 (24.6)	1200 (55.0)	2180 (100.0)	< 0.001
Heart Disease, n (%)	1087 (12.5)	219 (10.0)	227 (10.4)	298 (13.7)	343 (15.7)	< 0.001
FPG, mg/dl	102.42 (94.32,113.04)	99.36 (91.98, 107.82)	100.44 (93.60, 109.08)	101.88 (94.50, 111.78)	109.44 (99.54, 126.09)	< 0.001
HbA1c, %	5.1 (4.9, 5.4)	5.1 (4.8,5.4)	5.1 (4.9,5.4)	5.1 (4.9,5.4)	5.2 (4.9, 5.6)	< 0.001
TC, mg/dl	190.98 (167.78, 215.34)	185.18 (165.08, 208.38)	187.89 (165.46, 211.08)	192.91 (168.94, 216.88)	197.55 (172.81, 225.39)	< 0.001
TG, mg/dl	106.20 (75.23, 156.65)	61.95 (53.10, 71.69)	90.27 (78.77, 102.66)	126.56 (109.74, 145.14)	210.63 (172.58, 279.66)	< 0.001
HDL-C, mg/dl	49.48 (40.21, 59.92)	64.95 (57.22, 74.61)	53.35 (47.55, 59.92)	46.01 (40.98, 51.80)	36.34 (31.31, 41.75)	< 0.001
LDL-C, mg/dl	114.43 (93.94, 137.24)	109.02 (90.46,128.74)	117.53 (97.81,139.56)	122.17 (100.13, 144.30)	109.79 (85.44, 135.31)	< 0.001
GMS, n (%)						< 0.001
NGR	3467 (39.7)	1073 (49.2)	994 (45.6)	876 (40.1)	524 (24.0)	
Pre-DM	4044 (46.4)	954 (43.7)	971 (44.5)	1024 (46.9)	1095 (50.2)	
DM	1216 (13.9)	155 (7.1)	217 (9.9)	282 (12.9)	562 (25.8)	

Data were presented as mean±SD, median and interquartile range, or as n (%)

*AIP* Atherogenic index of plasma, *SBP* Systolic blood pressure, *DBP* Diastolic blood pressure, *BMI* Body mass index, *FPG* Fasting plasma glucose, *HbA1c* Hemoglobin A1c, *TC* Total cholesterol, *TG* Triglyceride, *HDL-C* High-density lipoprotein cholesterol, *LDL-C* Low-density lipoprotein cholesterol, *GMS* glucose metabolic states, *NGR* Normal glucose regulation, *Pre-DM* Prediabetes mellitus, *DM* Diabetes mellitus

**Table 2:** The HR (95% CI) of stroke according to AIP in the three Models

Categories	Event, *n* (%)	Model 1	Model 2	Model 3
		HR (95% CI) *P* value	HR (95% CI) *P* value	HR (95% CI) *P* value
Continuous variable per unit	734 (8.4)	2.65 (2.19–3.20) <0.001	2.19 (1.78–2.69) <0.001	1.90 (1.52–2.36) <0.001
Quartile				
Q1	111 (5.1)	Ref.	Ref.	Ref.
Q2	157 (7.2)	1.43 (1.12–1.82) 0.004	1.35 (1.05–1.72) 0.017	1.34 (1.05–1.71) 0.019
Q3	200 (9.2)	1.83 (1.45–2.31) <0.001	1.58 (1.24–2.01) <0.001	1.52 (1.19–1.93) 0.001
Q4	266 (12.2)	2.49 (1.99–3.10) <0.001	2.05 (1.62–2.58) <0.001	1.84 (1.45–2.34) <0.001

Model1: unadjusted

Model 2: adjusted for age, gender, marital status, drinking, smoking, residence, SBP, DBP, BMI

Model 3: Model 2 + adjusted for hypertension, heart disease, TC, FPG, HbA1c

**Table 3:** Association between AIP and the risk of stroke according to glucose metabolic states

Categories	Event, *n* (%)	Model1	Model2	Model3
		HR (95% CI) *P* value	HR (95% CI) *P* value	HR (95% CI) *P* value
NGR				
Continuous variable per unit	219 (6.3)	1.48 (0.93–2.36) 0.102	1.10 (0.67–1.81) 0.696	1.01 (0.61–1.67) 0.969
Quartile				
Q1	59 (5.5)	Ref.	Ref.	Ref.
Q2	64 (6.4)	1.18 (0.83–1.68) 0.37	1.08 (0.75–1.54) 0.681	1.06 (0.74–1.52) 0.752
Q3	59 (6.7)	1.23 (0.86–1.77) 0.26	1.07 (0.74–1.56) 0.721	1.03 (0.71–1.50) 0.873
Q4	37 (7.1)	1.30 (0.86–1.96) 0.22	1.03 (0.67–1.58) 0.907	0.96 (0.62–1.48) 0.849
Pre-DM				
Continuous variable per unit	352 (8.7)	2.73 (2.02–3.70) <0.001	2.44 (1.77–3.37) <0.001	2.37 (1.70–3.30) <0.001
Quartile				
Q1	47 (4.9)	Ref.	Ref.	Ref.
Q2	71 (7.3)	1.50 (1.04–2.17) 0.031	1.48 (1.02–2.14) 0.040	1.49 (1.03–2.16) 0.036
Q3	100 (9.8)	2.03 (1.43–2.86) <0.001	1.82 (1.27–2.60) 0.001	1.80 (1.26–2.57) 0.001
Q4	134 (12.2)	2.57 (1.85–3.59) <0.001	2.35 (1.66–3.33) <0.001	2.27 (1.60–3.23) <0.001
DM				
Continuous variable per unit	163 (13.4)	2.16 (1.56-3.00) <0.001	2.04 (1.44–2.90) <0.001	2.00 (1.36–2.93) <0.001
Quartile				
Q1	5 (3.2)	Ref.	Ref.	Ref.
Q2	22 (10.1)	3.22 (1.22–8.51) 0.018	3.03 (1.14–8.05) 0.026	3.08 (1.16–8.20) 0.024
Q3	41 (14.5)	4.71 (1.86–11.93) 0.001	4.18 (1.63–10.67) 0.003	3.95 (1.54–10.12) 0.004
Q4	95 (16.9)	5.62 (2.29–13.81) <0.001	4.87 (1.95–12.16) 0.001	4.58 (1.83–11.47) 0.001

Model 1: unadjusted

Model 2: adjusted for age, gender, marital status, drinking, smoking, residence, SBP, DBP, BMI

Model 3: Model 2 + adjusted for hypertension, heart disease, TC, FPG, HbA1c

**Table 4:** Subgroup and interaction analyses of the association between AIP and stroke

	Quartiles of AIP				*P* for interaction
	Q1	Q2	Q3	Q4	
Age					0.268
< 60 years (Case/Total)	1 (Ref.) 40/1282	1.55 (1.05–2.31) 66/1301	1.92 (1.32–2.81) 95/1314	2.30 (1.58–3.34) 140/1381	
≥ 60 years (Case/Total)	1 (Ref.) 71/900	1.23 (0.90–1.68) 91/881	1.27 (0.93–1.74) 105/868	1.57 (1.15–2.16) 126/800	
Gender					0.807
Female (Case/Total)	1 (Ref.) 50/1081	1.38 (0.97–1.97) 82/1208	1.45 (1.03–2.05) 106/1252	1.88 (1.34–2.64) 143/1201	
Male (Case/Total)	1 (Ref.) 61/1101	1.28 (0.91–1.81) 75/974	1.58 (1.13–2.21) 94/930	1.79 (1.27–2.52) 123/980	
BMI					0.011
< 24 kg/m^2^ (Case/Total)	1 (Ref.) 91/1749	1.22 (0.91–1.64) 92/1447	1.14 (0.83–1.56) 70/1095	1.99 (1.47–2.69) 99/834	
≥ 24 kg/m^2^ (Case/Total)	1 (Ref.) 20/433	1.75 (1.06–2.89) 65/735	2.14 (1.33–3.45) 130/1087	2.08 (1.30–3.34) 167/1347	
Residence					0.653
Rural (Case/Total)	1 (Ref.) 79/1596	1.46 (1.09–1.95) 114/1486	1.67 (1.25–2.23) 133/1380	2.03 (1.52–2.72) 161/1293	
Urban (Case/Total)	1 (Ref.) 32/586	1.06 (0.67–1.68) 43/696	1.19 (0.77–1.84) 67/802	1.53 (1.00-2.33) 105/888	
Hypertension					0.300
Yes (Case/Total)	1 (Ref.) 56/664	1.20 (0.85–1.70) 77/735	1.60 (1.16–2.21) 135/931	1.76 (1.27–2.42) 175/1047	
No (Case/Total)	1 (Ref.) 55/1518	1.53 (1.08–2.17) 80/1447	1.35 (0.93–1.96) 65/1251	1.99 (1.38–2.86) 91/1134	
GMS					0.031
NGR (Case/Total)	1 (Ref.) 59/1073	1.06 (0.74–1.52) 64/994	1.03 (0.71–1.50) 59/876	0.96 (0.62–1.48) 37/524	
Pre-DM (Case/Total)	1 (Ref.) 47/954	1.49 (1.03–2.16) 71/971	1.80 (1.26–2.57) 100/1024	2.27 (1.60–3.23) 134/1095	
DM (Case/Total)	1 (Ref.) 5/155	3.08 (1.16–8.20) 22/217	3.95 (1.54–10.12) 41/282	4.58 (1.83–11.47) 95/562	

Model adjusted for age, gender, marital status, drinking, smoking, residence, SBP, DBP, BMI, hypertension, heart disease, TC, FPG, HbA1c

**Fig. 2:**
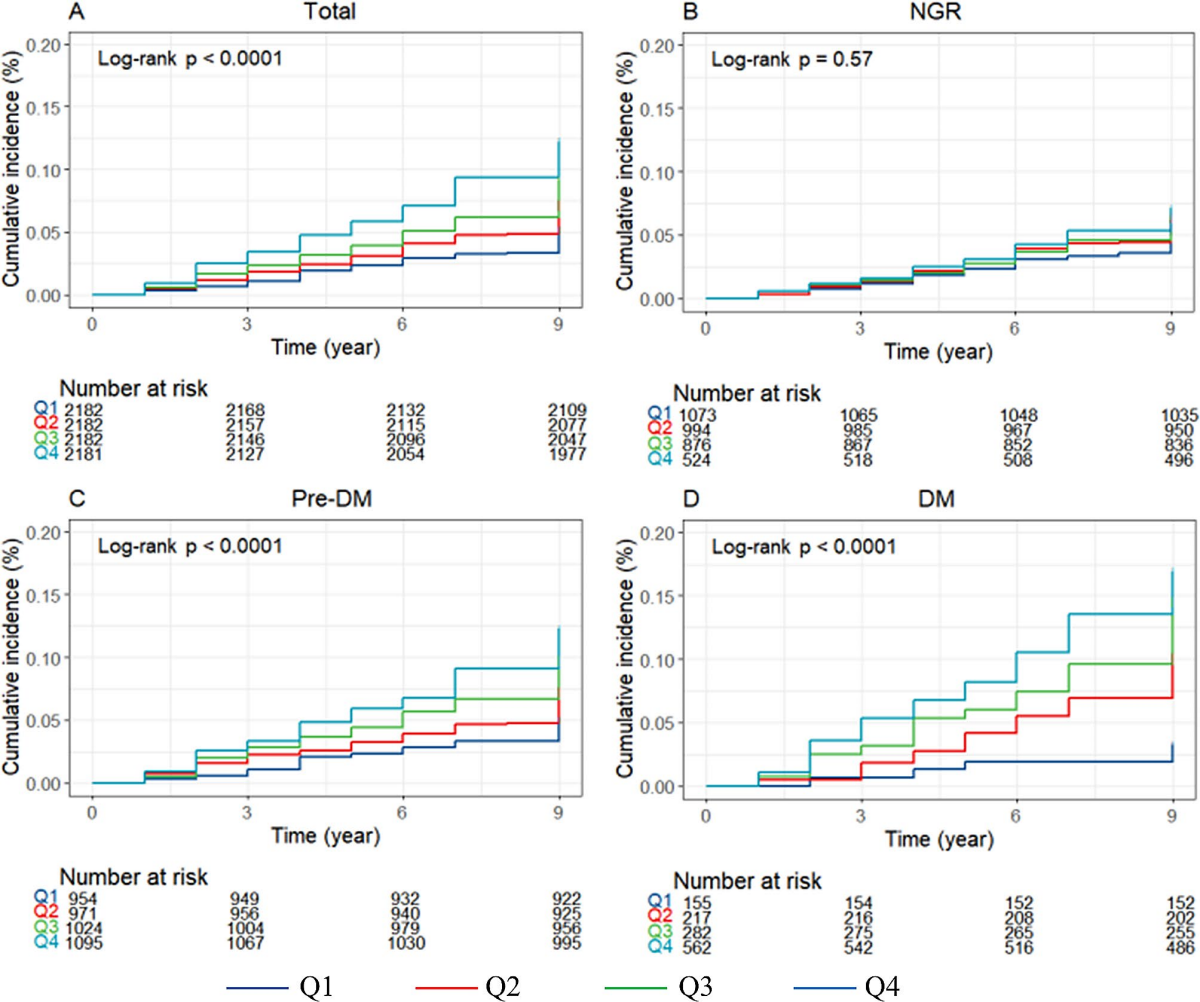
The Kaplan–Meier analysis for stroke was based on AIP quartiles for total participants (**A**), participants with NGR (**B**), participants with Pre-DM (**C**), and participants with DM (**D**)

**Fig. 3:**
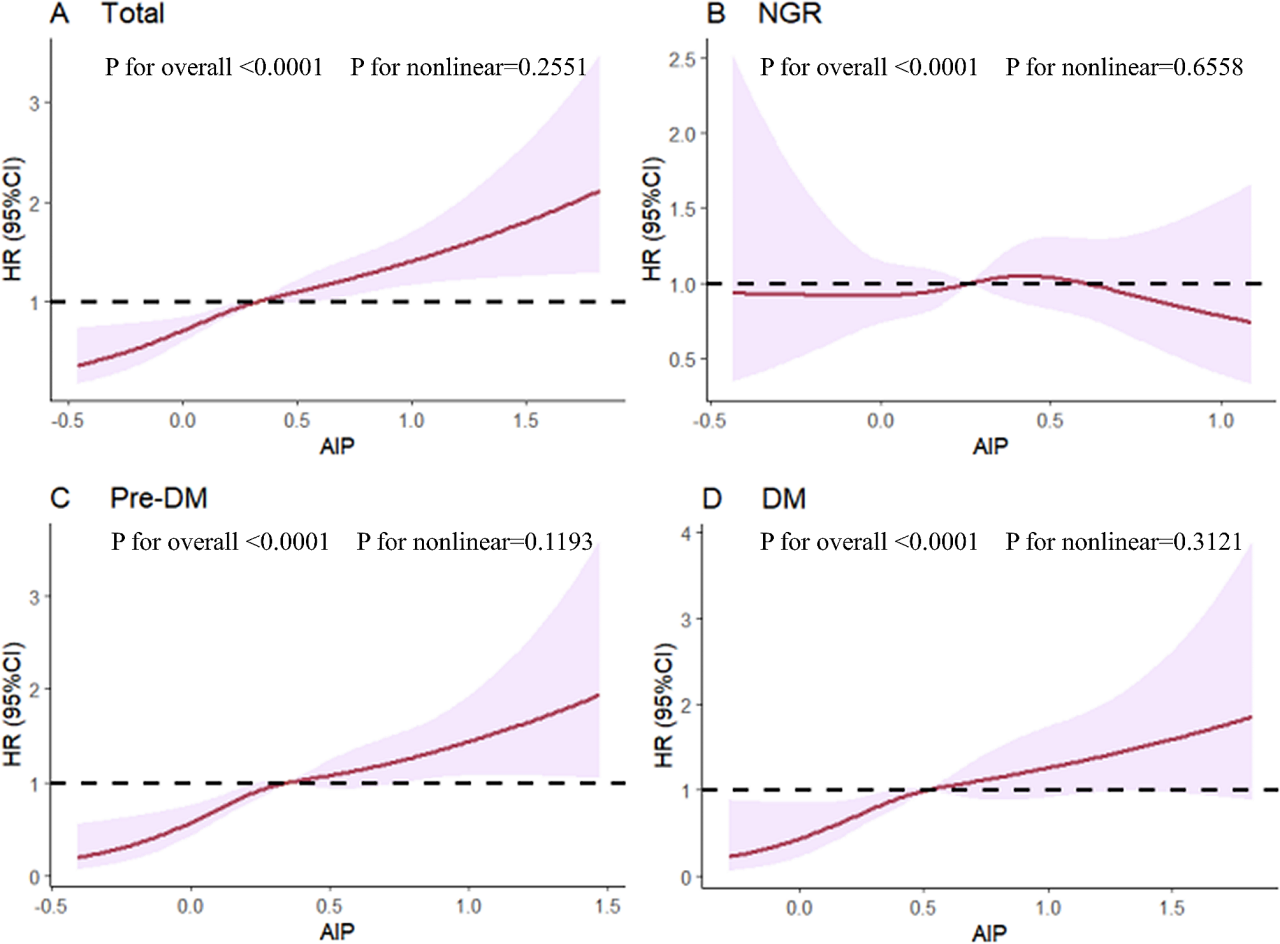
Association of AIP and the risk of stroke using a multivariable-adjusted restricted cubic spines model. Restricted cubic spline analysis has four knots at the 5th, 35th, 65th, and 95th percentiles of AIP. **A** total participants; **B** participants with NGR; **C** participants with Pre-DM. **D** participants with DM

### Predictive value of baseline AIP for the first stroke

During an average follow-up period of 8.72 years, 734 (8.4%) participants experienced their first stroke. According to the AIP quartiles, the incidences of stroke, from Q1 to Q4, were 5.65, 7.99, 10.18, 13.55 per 1000 person-years, respectively. Analysis of the Kaplan–Meier cumulative incidence curve revealed a gradual increase in stroke events from the Q1 to Q4 groups, with statistically significant difference observed (Fig. 2A log-rank test *P* < 0.0001). The Cox proportional hazard models confirmed a significant relationship between baseline AIP levels and new-onset stroke. Baseline AIP was analyzed as both a continuous variable and a categorical variable (quartiles). Following adjustment for potential confounding factors, per 1-unit increase in baseline AIP was associated with a 90% higher risk of stroke in Model 3 (HR 1.90, 95% CI 1.52–2.36). Furthermore, the risk of stroke showed an increasing trend across quartiles of AIP in Model 3 (HR 1.34, 95% CI 1.05–1.71 for Q2; HR 1.52, 95% CI 1.19–1.93 for Q3; HR 1.84, 95% CI 1.45–2.34 for Q4; p-trend 0.001, Table 2). Multivariable-adjusted restricted cubic splines analysis also demonstrated a significant dose-response relationship between the AIP as a continuous variable and the risk of stroke (P for overall trend < 0.001; P for nonlinear = 0.2551)(Fig. 3A).

### Associations between AIP and stroke regulated by individual glucose metabolic states

During the follow-up period, 219 (6.3%) participants with NGR, 352 (8.7%) participants with Pre-DM and 163 (13.4%) participants with DM were detected with the first stroke. The Kaplan–Meier curves (Fig. 2B–D) showed a significant difference in the cumulative incidence of stroke among Pre-DM and DM across the four AIP groups (*P* < 0.0001), while no significant difference was observed for NGR (*P* = 0.57). The results presented in Table 3 indicated that, in comparison to Q1, other AIP groups showed a significant association with an increased risk of stroke in individuals with Pre-DM and DM in Model 3. Specifically, for individuals with Pre-DM, HR were 1.49 (95% CI 1.03–2.16) for Q2, 1.80 (95% CI 1.26–2.57) for Q3, and 2.27 (95% CI 1.60–3.23) for Q4, with a p-trend of 0.001. In individuals with DM, HR were 3.08 (95% CI 1.16–8.20) for Q2, 3.95 (95% CI 1.54–10.12) for Q3, and 4.58 (95% CI 1.83–11.47) for Q4, with a p-trend of 0.001. However, no significant difference was found among AIP groups in individuals with NGR in the three Cox models (all p-values > 0.05). The restricted cubic splines analysis showed a notable increase in the risk of stroke in individuals with Pre-DM and DM as baseline AIP rises, demonstrating a linear relationship (Pre-DM: P for nonlinear = 0.1193; DM: P for nonlinear = 0.3121) (Fig. 3C–D). Conversely, the analysis did not reveal a significant dose-response correlation between AIP and the risk of stroke in individuals with NGR (Fig. 3B).

### Subgroup analysis

To further explore the association between baseline AIP and the first stroke event, we performed a subgroup analysis stratified by potential risk factors. As shown in Table 4, elevated AIP levels were associated with a higher incidence of stroke, which was consistent across different subgroups including age, gender, BMI, residence, and hypertension. Among individuals with Pre-DM and DM, increased AIP levels were linked to a higher stroke risk, whereas this association was not observed in the NGR groups. Significant interactions were noted between AIP and BMI (P value for interaction = 0.011) as well as between AIP and glucose metabolic status (P value for interaction = 0.031). However, no significant interactions were detected between AIP and other variables (all P values for interaction > 0.05).

### Sensitivity analyses

Several sensitivity analyses were carried out to assess the robustness of the results. After excluding 707 participants who fasted for less than 8 h prior to blood collection, Cox regression analyses generated consistent results with the primary analysis (see Additional file, Table S3, S4). Furthermore, there was no substantial change in the association between baseline AIP and stroke risk even after excluding participants with missing data for SBP, DBP, BMI and heart rate (see Additional file, Table S5, S6).

## Discussion

In the large national longitudinal survey cohort of middle-aged and elderly individuals, a significant correlation was elaborated between higher baseline AIP levels and an increased risk of a new-onset stroke. This association was particularly prominent in those with abnormal glucose metabolism, including Pre-DM and DM. The study suggested that baseline AIP might be a dependable biomarker for stratifying stroke risk. Maintaining a low AIP level might be beneficial for the primary prevention of stroke in individuals with abnormal glucose metabolism.

Dyslipidaemia and IR are features of the metabolic syndrome, both of which contribute to the risk of stroke [19–21]. The AIP is widely recognized as a reliable marker of dyslipidemia and atherosclerosis. Several studies have demonstrated that both baseline and cumulative AIP exposure are linked to cardiovascular diseases, particularly coronary artery disease [18, 22–24]. Notably, the impact of AIP on cardiovascular diseases may differ depending on an individual’s glucose metabolic state. Min et al. have found that individuals with abnormal glucose metabolism and higher AIP levels may have a greater risk of developing cardiovascular diseases [25]. In addition, higher levels of AIP have been found to be positively correlated with the risk of hypertension and non-alcoholic fatty liver disease, which is potentially influenced by the glucose metabolic states [26–28]. Interestingly, a cross-sectional study has demonstrated a strong association among elevated AIP levels, an increased risk of IR, and the onset of type 2 diabetes [6]. Elevation of TG or low HDL-C levels in the fasting or post-prandial state is observed in approximately half of individuals with type 2 diabetes, and is also frequently found in individuals with IR or impaired glucose tolerance [29, 30]. Research suggests that atherogenic dyslipidaemia is a significant risk factor for cardiovascular disease in individuals with abnormal glucose metabolism [31]. Further investigation is warranted to determine if AIP levels can serve as a marker for Pre-DM and DM. Recent research has explored the association between AIP and cerebrovascular disease, revealing that higher AIP levels are correlated with a higher incidence of atherosclerotic stenosis in the carotid and intracranial arteries [10, 32]. In the general population, increased baseline and cumulative AIP levels are associated with a greater risk of ischemic stroke [9, 11]. To the best of our knowledge, this study is the first to demonstrate the significant predictive value of baseline AIP level for stroke in individuals with glycemic dysregulation.

This study found that high baseline AIP levels were associated with new-onset stroke in individuals with Pre-DM and DM, which was consistent with previous reports that high AIP was associated with the risk and prognosis of stroke. Therefore, assessing AIP levels in middle-aged and elderly individuals with glycemic dysregulation would have clinical significance. Further studies are warranted to investigate the baseline levels of AIP, which is able to predict and identify stroke risk. Clinical studies have shown a significant correlation between stroke prognosis and the topography of stroke [33]. Consequently, it is valuable to investigate the potential impact of baseline AIP on the location of cerebrovascular topography in stroke patients. Lowering LDL-cholesterol has been traditionally believed to be beneficial in preventing overall stroke, with statins being commonly used for this purpose [34]. However, a multicenter clinical trial shows that patients with high triglycerides levels are accompanied with a high risk of ischemic stroke, despite statin therapy [35]. Recent data also advocated for lowering triglycerides as a strategy to prevent stroke [36]. Therefore, it is plausible to consider that lowering triglycerides, which is equivalent to lowering AIP levels, contributes to stroke prevention.

Although the mechanisms underlying the relationship between AIP and stroke remain unclear, several possible explanations have been proposed. Firstly, triglycerides appear to be related to vascular inflammation and subclinical atherosclerosis. Raised serum triglycerides can potentiate inflammatory responses in vascular endothelial cells and vascular smooth muscle cells, especially in diabetic patients [37, 38]. Emerging evidence indicates that triglycerides-rich lipoproteins like chylomicrons and very low-density lipoproteins may play a role in atherosclerotic lesion formation [39, 40]. Besides, HDL particles exhibit various vasoprotective properties, such as reducing cellular death, dampening inflammatory response, and shielding against pathological oxidation [41]. Therefore, it can be inferred that as AIP increase, higher levels of triglycerides lead to more significant damage to vascular structure and function, while lower levels of HDL offer less protection to the vasculature. Secondly, the level of AIP has been investigated to be closely associated with traditional risk factors for stroke, including BMI, hypertension, diabetes, dyslipidemia, and heart disease. The results of the present study are consistent with these studies. Elevated AIP levels may interact with other cerebrovascular risk factors and potentially exacerbate the progression of stroke. However, further research is necessary to fully understand the underlying mechanisms.

## Strengths and limitations

This is the largest population-based investigation into the association between AIP and stroke among middle-aged and elderly individuals with glycemic dysregulation. The data was obtained from a high-quality, nationally representative longitudinal survey of the middle-aged and elderly population across various regions of China, including urban and rural areas. In order to obtain robust results, we included potential confounders to exclude interference in the results. With nearly a decade of follow-up, our analysis indicated that baseline AIP was a reliable predictor of stroke in middle-aged and elderly individuals with dysglycaemia. Moreover, since standard assay for TG and HDL-C are widely used in clinical practice and it is straightforward to calculate AIP from TG and HDL-C, it is sensible to recommend AIP as an efficient and convenient indicator for assessing the risk of stroke.

However, there are several limitations of the study that require consideration. Firstly, baseline medications such as antihypertensive, lipid-lowering, and glucose-lowering medications were not taken into account in the Cox proportional hazards model, potentially impacting the results. Secondly, while the diagnostic criteria for glucose metabolism status have been clearly defined, it remains a slight chance of misclassification for participants with borderline glycaemic status. Thirdly, some participants from the CHARLS cohort were excluded from this research due to strict exclusion criteria, resulting in a limited number of participants included. This may have introduced attrition bias. Fourthly, the study was based on middle-aged and elderly Chinese, and further validation in other ethnic and age groups is needed. Fifthly, this study focused on the impact of baseline AIP level and did not examine the longitudinal changes in AIP during the follow-up period. Sixthly, due to a lack of data on stroke subtypes in CHARLS, the study was unable to assess the effect of AIP on ischemic or hemorrhagic stroke, respectively. Finally, despite efforts to control for confounding variables, there is a possibility that some confounders were not accounted for. Further investigations are needed to verify our findings in other large cohort studies

## Conclusions

In the pursuit of primary prevention strategies to reduce stroke incidence, this longitudinal prospective study found that baseline high AIP levels in individuals with glycemic dysregulation might indicate subgroups at a higher risk of developing stroke, particularly among individuals under 60 years old with a BMI ≥ 24 kg/m^2^ residing in rural areas. However, the levels of AIP in middle-aged and older adults without dysglycaemia did not affect the occurrence of stroke.

### Electronic supplementary material

Below is the link to the electronic supplementary material.

Additional file

## Data Availability

The datasets generated and/or analysed during the current study are available in the China Health and Retirement Longitudinal Study repository, which can be accessed online at http://charls.pku.edu.cn. You can register as a CHARLS user to access all published data by following the necessary procedure.

## Abbreviations

AIP: Atherogenic index of plasma
SBP: Systolic blood pressure
DBP: Diastolic blood pressure
BMI: Body mass index
FPG: Fasting plasma glucose
HbA1c: Hemoglobin A1c
TC: Total cholesterol
TG: Triglyceride
HDL-C: High-density lipoprotein cholesterol
LDL-C: Low-density lipoprotein cholesterol
GMS: glucose metabolic states
NGR: Normal glucose regulation
Pre-DM: Prediabetes mellitus
DM: Diabetes mellitus
