# Supplementary material for: Association between atherogenic index of plasma and new-onset stroke in individuals with different glucose metabolism status: insights from CHARLS
Source: Cardiovasc Diabetol. 2024 Jun 21;23:215. doi: 10.1186/s12933-024-02314-y (PMC11193183; doi:10.1186/s12933-024-02314-y)
Supplement: Supplementary file 1 — Additional file [file 12933_2024_2314_MOESM1_ESM.docx]

**Table S1. Distribution of missing data.**

| Characteristics | No. of missing values | Percent(%) | Disposition |
| --- | --- | --- | --- |
| Age | 0 | 0 | - |
| Gender | 0 | 0 | - |
| Education | 0 | 0 | - |
| Marital status | 0 | 0 | - |
| Living place | 0 | 0 | - |
| Drinking | 0 | 0 | - |
| Smoking | 0 | 0 | - |
| SBP | 1189 | 13.6 | Multiple imputation |
| DBP | 1189 | 13.6 | Multiple imputation |
| Heart Rate | 1189 | 13.6 | Multiple imputation |
| BMI | 1216 | 13.9 | Multiple imputation |
| Hypertension | 0 | 0 | - |
| Diabetes | 0 | 0 | - |
| Dyslipidemia | 0 | 0 | - |
| Heart disease | 0 | 0 | - |
| FPG | 0 | 0 | - |
| HbA1c | 0 | 0 | - |
| TC | 0 | 0 | - |
| TG | 0 | 0 | - |
| HDL-C | 0 | 0 | - |
| LDL-C | 14 | 0.2 | Multiple imputation |
| AIP | 0 | 0 | - |

Abbreviations: SBP, Systolic blood pressure; DBP, Diastolic blood pressure; BMI, Body mass index; FPG, Fasting plasma glucose; HbA1c, Hemoglobin A1c; TC, Total cholesterol; TG, Triglyceride; HDL-C, High-density lipoprotein cholesterol; LDL-C, Low-density lipoprotein cholesterol; AIP, Atherogenic index of plasma;

**Table S2. Baseline characteristics of excluded and included participants.**

| **Characteristics** | **Total** | **Included** | **Excluded** | **P value** |
| --- | --- | --- | --- | --- |
| No. of participants | 17708 | 8727 | 8981 |  |
| Age, years | 58.50±10.17 | 58.04±8.75 | 59.0+11.39 | <0.001 |
| Female, n (%) | 9228(52.1) | 4742(54.3) | 4486(50.0) | <0.001 |
| SBP, mmHg | 130.35±21.84 | 129.40±20.01 | 131.33±22.40 | <0.001 |
| DBP, mmHg | 75.76±12.40 | 75.46±11.37 | 76.15±12.69 | 0.001 |
| Heart rate, bpm | 72.30±10.84 | 71.95±9.84 | 72.74±11.20 | <0.001 |
| BMI, kg/m2 | 23.43±3.73 | 23.52±3.35 | 23.32±3.82 | <0.001 |
| Rural residence, n (%) | 10537(59.5) | 5755(65.9) | 4782(53.2) | <0.001 |
| Married, n (%) | 14170(80.2) | 7337(84.1) | 6852(76.4) | <0.001 |
| Education, n (%) |  |  |  | <0.001 |
| Junior high school and below | 15545(87.9) | 7839(89.8) | 7722(86.0) |  |
| Senior high school | 1793(10.1) | 788(9.0) | 1005(11.2) |  |
| Tertiary | 354(2.0) | 100(1.1) | 254(2.8) |  |
| Smoking, n (%) |  |  |  | <0.001 |
| Never | 10619(60.5) | 5400(61.9) | 5308(59.1) |  |
| Former | 2069(11.8) | 793(9.1) | 1293(14.4) |  |
| Current | 4871(27.7) | 2534(29.0) | 2380(26.5) |  |
| Drinking, n (%) |  |  |  | 0.014 |
| Never | 10343(58.9) | 5143(58.9) | 5291(58.9) |  |
| Former | 1443(8.2) | 668(7.7) | 787(8.8) |  |
| Current | 5767(32.9) | 2916(33.4) | 2903(32.3) |  |
| Hypertension, n (%) | 6732(38.5) | 3377(38.7) | 3515(39.1) | 0.574 |
| Diabetes, n (%) | 2381(13.7) | 1216(13.9) | 981(10.9) | <0.001 |
| Dyslipidemia, n (%) | 6326(36.5) | 4230(48.5) | 2160(24.1) | <0.001 |
| Heart Disease, n (%) | 2368(13.6) | 1087(12.5) | 1309(14.6) | <0.001 |
| FPG, mg/dl | 102.42(94.32, 113.76) | 102.42(94.32,113.04) | 102.78 (94.50, 115.74) | 0.095 |
| HbA1c, % | 5.1(4.9,5.4) | 5.1(4.9,5.4) | 5.1(4.8,5.4) | <0.001 |
| TC, mg/dl | 189.82(166.62,215.34) | 190.98(167.78,215.34) | 186.73(161.60,214.18) | <0.001 |
| TG, mg/dl | 106.20(75.23, 156.65) | 106.20(75.23,156.65) | 106.20(75.23, 155.76) | 0.726 |
| HDL-C, mg/dl | 49.10(39.82, 59.54) | 49.48(40.21,59.92) | 48.33(39.43,58.76) | <0.001 |
| LDL-C, mg/dl | 114.05(92.78,136.86) | 114.43(93.94,137.24) | 111.34(88.92, 134.92) | <0.001 |

Data were presented as mean±SD, median and interquartile range, or as n (%)

Abbreviations: AIP, Atherogenic index of plasma; SBP, Systolic blood pressure; DBP, Diastolic blood pressure; BMI, Body mass index; FPG, Fasting plasma glucose; HbA1c, Hemoglobin A1c; TC, Total cholesterol; TG, Triglyceride; HDL-C, High-density lipoprotein cholesterol; LDL-C, Low-density lipoprotein cholesterol;

**Table S3 The association of AIP with stroke after excluding individuals with less than 8 hours of fasting**

| Categories | Event, n(%) | Model1 | Model2 | Model3 |
| --- | --- | --- | --- | --- |
|  |  | HR (95% CI) P value | HR (95% CI) P value | HR (95% CI) P value |
| Continuous variable per unit | 680(8.5) | 2.65(2.17-3.24) <0.001 | 2.22(1.79-2.76) <0.001 | 1.94(1.54-2.45) <0.001 |
| Quartile |  |  |  |  |
| Q1 | 103(5.0) | Ref. | Ref. | Ref. |
| Q2 | 150(7.4) | 1.50(1.17-1.93) 0.001 | 1.43(1.11-1.84) 0.006 | 1.42(1.10-1.82) 0.007 |
| Q3 | 189(9.4) | 1.91(1.50-2.43) <0.001 | 1.65(1.29-2.11) <0.001 | 1.59(1.24-2.04) 0.001 |
| Q4 | 238(12.3) | 2.56(2.03-3.23) <0.001 | 2.12(1.66-2.71) <0.001 | 1.91(1.49-2.45) <0.001 |

Model1: unadjusted

Model 2: adjusted for age, gender, marital status, drinking, smoking, residence, SBP, DBP, BMI

Model 3: Model 2+adjusted for hypertension, heart disease, TC, FPG, HbA1c

**Table S4 Association between AIP and the risk of stroke according to glucose metabolic states after excluding individuals with less than 8 hours of fasting**

| Categories | Event, n(%) | Model1 | Model2 | Model3 |
| --- | --- | --- | --- | --- |
|  |  | HR (95% CI) P value | HR (95% CI) P value | HR (95% CI) P value |
| **NGR** |  |  |  |  |
| Continuous variable per unit | 208(6.4) | 1.50(0.92-2.44) 0.103 | 1.10(0.66-1.85) 0.710 | 1.02(0.61-1.72) 0. 931 |
| Quartile |  |  |  |  |
| Q1 | 56(5.5) | Ref. | Ref. | Ref. |
| Q2 | 63(6.7) | 1.23(0.86-1.76) 0.262 | 1.12(0.78-1.62) 0.529 | 1.11(0.77-1.60) 0.585 |
| Q3 | 55(6.8) | 1.24(0.85-1.79) 0.264 | 1.07(0.73-1.57) 0.740 | 1.04(0.71-1.54) 0.826 |
| Q4 | 34(7.2) | 1.33(0.87-2.03) 0.194 | 1.03(0.66-1.61) 0.895 | 0.97(0.62-1.52) 0.902 |
| **Pre-DM** |  |  |  |  |
| Continuous variable per unit | 328(8.7) | 2.93(2.14-4.02) <0.001 | 2.72(1.94-3.81) <0.001 | 2.63(1.85-3.73) <0.001 |
| Quartile |  |  |  |  |
| Q1 | 42(4.6) | Ref. | Ref. | Ref. |
| Q2 | 67(7.4) | 1.62(1.10-2.38) 0.014 | 1.60(1.09-2.36) 0.017 | 1.60(1.09-2.36) 0.017 |
| Q3 | 97(10.1) | 2.25(1.56-3.23) <0.001 | 2.02(1.39-2.93) <0.001 | 1.98(1.37-2.88) <0.001 |
| Q4 | 122(12.2) | 2.74(1.93-3.89) <0.001 | 2.51(1.74-3.63) <0.001 | 2.41(1.66-3.50) <0.001 |
| **DM** |  |  |  |  |
| Continuous variable per unit | 144(14.0) | 1.94(1.37-2.75) <0.001 | 1.88(1.29-2.73) 0.001 | 1.82(1.20-2.75) 0.005 |
| Quartile |  |  |  |  |
| Q1 | 5(3.6) | Ref. | Ref. | Ref. |
| Q2 | 20(11.4) | 3.21(1.20-8.55) 0.020 | 3.12(1.16-8.38) 0.024 | 3.16(1.18-8.51) 0.022 |
| Q3 | 37(14.9) | 4.31(1.69-10.95) 0.002 | 3.95(1.53-10.19) 0.005 | 3.82(1.48-9.85) 0.006 |
| Q4 | 82(17.7) | 5.26(2.13-12.98) <0.001 | 4.85(1.93-12.21) 0.001 | 4.57(1.81-11.53) 0.001 |

Model1: unadjusted

Model 2: adjusted for age, gender, marital status, drinking, smoking, residence, SBP, DBP, BMI

Model 3: Model 2+adjusted for hypertension, heart disease, TC, FPG, HbA1c

**Table S5 The association of AIP with stroke after excluding individuals with missing data for SBP, DBP, BMI and Heart rate**

| Categories | Event, n(%) | Model1 | Model2 | Model3 |
| --- | --- | --- | --- | --- |
|  |  | HR (95% CI) P value | HR (95% CI) P value | HR (95% CI) P value |
| Continuous variable per unit | 634(8.5) | 2.75(2.25-3.37) <0.001 | 2.26(1.82-2.82) <0.001 | 2.01(1.59-2.54) <0.001 |
| Quartile |  |  |  |  |
| Q1 | 88(4.6) | Ref. | Ref. | Ref. |
| Q2 | 147(7.9) | 1.73(1.33-2.26) <0.001 | 1.63(1.25-2.12) <0.001 | 1.62(1.24-2.12) <0.001 |
| Q3 | 178(9.5) | 2.10(1.62-2.71) <0.001 | 1.79(1.37-2.32) <0.001 | 1.72(1.32-2.24) <0.001 |
| Q4 | 221(12.1) | 2.73(2.13-3.50) <0.001 | 2.22(1.71-2.88) <0.001 | 2.01(1.54-2.62) <0.001 |

Model1: unadjusted

Model 2: adjusted for age, gender, marital status, drinking, smoking, residence, SBP, DBP, BMI

Model 3: Model 2+adjusted for hypertension, heart disease, TC, FPG, HbA1c

**Table S6 Association between AIP and the risk of stroke according to glucose metabolic states after excluding individuals with missing data for SBP, DBP, BMI and Heart rate**

| Categories | Event, n(%) | Model1 | Model2 | Model3 |
| --- | --- | --- | --- | --- |
|  |  | HR (95% CI) P value | HR (95% CI) P value | HR (95% CI) P value |
| **NGR** |  |  |  |  |
| Continuous variable per unit | 191(6.4) | 1.78 (1.08-2.93) 0.024 | 1.32(0.78-2.24) 0.297 | 1.23(0.72-2.09) 0.448 |
| Quartile |  |  |  |  |
| Q1 | 45(4.7) | Ref. | Ref. | Ref. |
| Q2 | 62(7.3) | 1.55(1.06-2.28) 0.025 | 1.42(0.96-2.10) 0.076 | 1.42(0.96-2.09) 0.080 |
| Q3 | 52(6.9) | 1.46(0.98-2.18) 0.062 | 1.27(0.84-1.91) 0.264 | 1.22(0.81-1.85) 0.342 |
| Q4 | 32(7.3) | 1.55(0.98-2.43) 0.060 | 1.22(0.76-1.95) 0.420 | 1.15(0.71-1.85) 0.571 |
| **Pre-DM** |  |  |  |  |
| Continuous variable per unit | 304(8.8) | 2.57(1.85-3.57) <0.001 | 2.29(1.61-3.26) <0.001 | 2.26(1.57-3.24) <0.001 |
| Quartile |  |  |  |  |
| Q1 | 40(4.8) | Ref. | Ref. | Ref. |
| Q2 | 65(7.9) | 1.65(1.11-2.45) 0.013 | 1.60(1.08-2.38) 0.020 | 1.61(1.08-2.39) 0.019 |
| Q3 | 93(10.5) | 2.23(1.54-3.23) <0.001 | 1.97(1.35-2.89) <0.001 | 1.94(1.33-2.85) 0.001 |
| Q4 | 106(11.6) | 2.48(1.73-3.57) <0.001 | 2.22(1.52-3.26) <0.001 | 2.17(1.48-3.19) <0.001 |
| **DM** |  |  |  |  |
| Continuous variable per unit | 139(13.4) | 2.43(1.72-3.41) <0.001 | 2.31(1.59-3.34) <0.001 | 2.36(1.56-3.58) <0.001 |
| Quartile |  |  |  |  |
| Q1 | 3(2.2) | Ref. | Ref. | Ref. |
| Q2 | 20(10.5) | 4.91(1.46-16.52) 0.010 | 4.60(1.36-15.56) 0.014 | 4.65(1.37-15.76) 0.013 |
| Q3 | 33(13.7) | 6.48(1.99-21.12) 0.002 | 5.69(1.73-18.73) 0.004 | 5.34(1.62-17.61) 0.006 |
| Q4 | 83(17.8) | 8.70(2.75-27.53) <0.001 | 7.44(2.32-23.89) 0.001 | 6.95(2.16-22.41) 0.001 |

Model1: unadjusted

Model 2: adjusted for age, gender, marital status, drinking, smoking, residence, SBP, DBP, BMI

Model 3: Model 2+adjusted for hypertension, heart disease, TC, FPG, HbA1c
